# Supplementary material for: Usefulness of Vaccine Adverse Event Reporting System for Machine-Learning Based Vaccine Research: A Case Study for COVID-19 Vaccines
Source: Int J Mol Sci. 2022 Jul 26;23(15):8235. doi: 10.3390/ijms23158235 (PMC9368306; doi:10.3390/ijms23158235)
Supplement: Supplementary file 1 [file ijms-23-08235-s001.zip › ijms-1793654-supplementary.pdf]

Usefulness of Vaccine Adverse Event Reporting System for machine learning based vaccine research: A case study for Covid-19 vaccines

James Flora[1], Wasiq Khan[2], Jennifer Jin[1], Daniel Jin[3], Abir Hussain[4],
Khalil Dajani[1] & Bilal Khan[1,5,*]

(1) Department of Computer Science and Engineering, California State University San Bernardino, 5500 University Parkway, San Bernardino, California 92407 USA.

(2) School of Computer Science and Mathematics, Liverpool John Moores University; Liverpool, United Kingdom

(3) Division of Vascular & Interventional Radiology, Department of Radiology, Loma Linda University Medical Center, Loma Linda, CA

(4) Department of Electrical Engineering, University of Sharjah, Sharjah, UAE

(5) Institute of the Environment and Sustainability, University of California Los Angeles; Los Angeles, California 90095, USA

(*) Author(s) to whom correspondence should be addressed.

Submitted to: International Journal of Molecular Sciences

Date: 6/13/2022

S1. Introduction

VAERS data sets have been used in various studies for recommendations and proactive strategies for regulatory bodies (CDC and FDA) [1,2,11–18,3–10]. For example, named entity recognition (NER) was carried out to extract nervous system disorder-related events from vaccine safety reports via deep learning approaches in [19]. In this study, 91 reports of GBS related influenza vaccine safety were extracted from VAERS for the period 1990 – 2016 and carefully annotated with major entities related to nervous system disorders including *investigation*, *nervous AE*, *other AE*, *procedure*, *social circumstance*, and *temporal expression*. The initial pool consisted of 1,849 reports which were then filtered to remove the reports with a short length of text (e.g., less than 1100 characters). Subsequently, 9 reports were further removed after manual review that contained duplicate texts with other reports, resulting into 91 reports in total for the analysis. Although careful annotations and manual reviews were performed in the above studies [3,19], application of data provenance, cleaning and normalization techniques (as proposed in the present study) or the datasets that were utilized for model development were not made publicly available to verify duplicates, spelling/grammar errors, outliers or other data normalization/standardization concerns. A ML and natural language processing approach was developed to identify COVID-19 vaccine AEs (VAE) from Twitter data [15]. Based on COVID-19 vaccine-related tweets (1 December 2020–1 August 2021), a ML based pipeline was proposed to identify tweets containing personal experiences with COVID-19 vaccinations and to extract and normalize VAE-related entities, including dose(s); vaccine types (Pfizer, Moderna, and Janssen); and symptom(s) from tweets. Data included 66,499 VAE mentions from a total of 111,229 tweets based on which the top 3 most frequently discussed AEs for each of the three COVID-19 vaccines were identified (i.e., *Sore to touch, fatigue,* and *headache*). Furthermore, the top 10 AEs reported in VAERS were visualized

and compared with the results from the analysis of Twitter-based data. A detailed sequence of data preprocessing and cleaning along with the size of VAERS dataset was not provided. It is noted that, although VAERS dataset was used for exploratory analysis and comparison of the top reported AEs, results (as demonstrated in the proposed study) can be misleading without employing specific data preprocessing practices, thus leading to potential model discrepancies.

Another study emphasized on 23,092 VAERS reports of U.S. patients on Live-attenuated HZ vaccine (zoster vaccine live, ZVL) for the analytic period of May 2006–January 2015, of which only 972 (4%) reports were classified as serious for the analysis, including 74 deaths [18]. Among persons aged ≥50 years who received ZVL alone, *injection-site reactions*, *HZ* and *rash* were the most commonly reported non-serious AEs while *HZ, pain, rash, dyspnoea* and *pyrexia* were the most commonly reported serious AEs. In their analysis, Empirical Bayesian data mining was also carried out, which did not indicate new or unexpected safety signals. The study provided identification of the most commonly reported AEs along with the acknowledgment by the authors that VAERS data is not suitable for causal analysis. Brief description of the data cleaning, preprocessing and verification strategies were reported for statistical analysis and model development pipeline.

In another study, the effectiveness of Covid-19 vaccines (Pfizer-BioNTech and Moderna) was analyzed based on 4,417 samples of VAERS data (i.e., 4,009 and 708 data samples for model training and testing, respectively) while considering certain demographic factors such as age, gender, and state of living (to understand the impact of food habits and living conditions) [16]. Initial size of the dataset in the study before applying data preprocessing steps comprised of 9,287 samples and various ML techniques (i.e., logistic regression, Adaboost, decision trees and random forests) were then applied with DIED column set as a target variable and demonstrated the

accuracy of these models to be >97%. It is noted that VAERS allows more than 5 symptoms by splitting those entries as separate rows in the data (with same VAERS ID), where data preprocessing steps in their study indicated the selection of only first 5 symptoms, potentially leading to information loss, incomplete evidence for each report and thus potentially inducing bias in ML models. Statistical analysis was conducted on VAERS data in another study to analyze AE profiles induced by hepatitis A vaccine (Havrix), hepatitis B vaccine (Engerix-B) and hepatitis A and B combination vaccine (Twinrix) [17]. The study identified 46, 69 and 82 AEs significantly associated with Havrix, Engerix-B and Twinrix, respectively. Additionally, hierarchical classification proposed these AEs to be related to behavioral and neurological conditions, immune system, and investigation results. The analysis utilized a logistic regression model accompanied by Markov Chain Monte Carlo (MCMC) sampling demonstrating 13 AEs (e.g., *hepatosplenomegaly*) identified to result from VVI synergistic effects.

S2. Materials and Methods

S2.1. Compilation of VAERS data, preprocessing, and exploration: Datasets compiled from VAERS without removing duplicates consisted of 905,976 data samples. Children reports from VAERS were distributed as 11,262, 956, 260, and 11 for Pfizer-BioNTech, Moderna, Janssen, and unknown, respectively. Discretization of the age attribute was initially for the groups of {≥16, and <16} due to the approval status for each vaccine (i.e., Pfizer-BioNTech and Moderna were first approved for adults only). Textual data from VAERS symptoms fields for exploratory analysis and correlation assessment via SOMs (**Section S2.2.1**) were transformed into structured corpus of columns and rows such that the rows and columns represent the number of data samples and reported AEs within the dataset, respectively. Additionally, for the identification of many-to-many

relationships among the AEs via ARM, symptom data were further extracted in the form of rows representing the occurrence of AEs as comma separated transactions. Data for psychological effects from online survey was stored as binary indicator for each column that was used to explore their relationships via SOMs.

Table S1: Number of VAERS reports categorized with respect to the age group and gender along with their percentage (from a total of 905,976 VAERS reports) per three vaccine producers. Below statistics were reported prior to the merger of duplicate rows.

	Vaccine					
	Pfizer-BioNTech		Moderna		Janssen	
**Age Group (years)**	**Male**	**Female**	**Male**	**Female**	**Male**	**Female**
5 - 11	713 (0.63%)	721 (0.28%)	23 (0.02%)	32 (0.01%)	6 (0.02%)	5 (0.01%)
12 - 15	6,623 (5.88%)	6,470 (2.48%)	439 (0.43%)	485 (0.17%)	136 (0.52%)	114 (0.25%)
16 - 18	5,101 (4.53%)	5,889 (2.25%)	2,903 (2.85%)	3,936 (1.41%)	874 (3.37%)	914 (1.99%)
19 - 30	14,281 (12.67%)	33,680 (12.89%)	11,404 (11.18%)	31,011 (11.12%)	5,450 (21.03%)	7,601 (16.58%)
31 - 50	31,738 (28.16%)	98,732 (37.77%)	26,468 (25.95%)	94,362 (33.84%)	9,260 (35.73%)	18,792 (41.00%)
51 - 65	25,866 (22.95%)	67,912 (25.98%)	26,289 (25.77%)	75,658 (27.14%)	7,124 (27.49%)	13,370 (29.17%)
66+	28,400 (25.19%)	47,968 (18.35%)	34,469 (33.79%)	73,328 (26.30%)	3,066 (11.83%)	5,037 (10.99%)
**Total**	112,722	261,372	101,995	278,812	25,916	45,833

Note: The number of samples per vaccine manufacturer and their percentages were calculated using clean data by removing those samples where any of the four attributes {age, gender, vaccine manufacturer, and symptom} were listed as "unknown"

Table S2: Summary of the 20 most commonly reported AEs in VAERS reports and online survey data per three vaccine producers

Effects		Vaccine manufacturer				
**VAERS**	**Survey Data**	**Pfizer-BioNTech**	**Pfizer-BioNTech survey data points**	**Moderna**	**Moderna survey data points**	**Janssen**
Headache	Headache	50,235 (12.55%)	44 (35.77%)	53,440 (13.14%)	31 (46.97%)	15,259 (18.34%)
Pyrexia	Aches	39,231 (9.80%)	53 (43.09%)	49,129 (12.08%)	44 (66.67%)	12,832 (15.42%)
Fatigue	Tired	42,980 (10.73%)	78 (63.41%)	46,285 (11.38%)	48 (72.73%)	10,421 (12.52%)
Chills	Chills	33,256 (8.31%)	54 (43.90%)	42,490 (10.45%)	44 (66.67%)	11,013 (13.23%)
Pain	Pain in muscle	36,006 (8.99%)	49 (39.84%)	39,074 (9.61%)	34 (51.52%)	10,443 (12.55%)
Dizziness	Dizziness	32,895 (8.22%	2 (1.63%)	26,645 (6.55%)	13 (19.70%)	8,109 (9.74%)
Nausea	Nausea	29,248 (7.31%)	13 (10.57%)	30,091 (7.40%)	11 (16.67%)	7,831 (9.41%)
Pain in Extremity	NA	25,730 (6.43%)	NA	32,703 (8.04%)	NA	6,030 (7.25%)
Myalgia	NA	15,726 (3.93%)	NA	21,271 (5.23%)	NA	4,067 (4.89%)
Arthralgia	NA	18,311 (4.57%)	NA	18,431 (4.53%)	NA	3,508 (4.22%)
Injection site pain	NA	17,101 (4.27%)	NA	31,175 (7.67%)	NA	3,620 (4.35%)
Dyspnoea	NA	19,144 (4.78%)	NA	15,245 (3.75%)	NA	3,903 (4.69%)
Rash	Itchy Skin/Rash	14,801 (3.70%)	1 (0.81%)	18,277 (4.49%)	2 (3.03%)	2,197 (2.64%)
Pruritus	NA	12,388 (3.09%)	NA	18,031 (4.43%)	NA	1,454 (1.75%)
Injection site erythema	NA	4,794 (1.20%)	NA	28,029 (6.89%)	NA	600 (0.72%)
Asthenia	Strange Feeling	11,859 (2.96%)	10 (8.13%)	12,688 (3.12%)	15 (22.73%)	2,871 (3.45%)
Vomiting	Vomiting	11,723 (2.93%)	NA	11,892 (2.92%)	2 (3.03%)	3,018 (3.63%)
Injection site swelling	Enlarged lymph nodes	4,944 (1.23%)	1 (0.81%)	21,700 (5.34%)	1 (1.52%)	742 (0.89%)
Diarrhoea	NA	10,465 (2.61%)	NA	10,122 (2.49%)	NA	2,021 (2.43%)
Erythema	NA	6,433 (1.61%)	NA	14,459 (3.56%)	NA	838 (1.01%)

Note: Numbers in the table indicate the number of VAERS samples that reported corresponding adverse event and the percentage shows the percent of all VAERS reports that were vaccinated by the given vaccine manufacturer. Survey data for Janssen were not available.

S2.2. Correlation analysis of AEs based on age groups and pre-existing conditions: Exploratory analysis can be valuable for assessing the quality of available data and identifying suitable recommendations for data quality improvement. Exploratory analysis including bipartite graphs and unsupervised ML approaches (association rule mining, self-organizing maps, hierarchical clustering) were applied on unlabeled Covid-19 vaccine data, collected from VAERS and online personalized survey where the endpoints (i.e., reported effects and their causes) were analyzed to explore the relationships among various AEs and subsequently among the reported pre-existing conditions (i.e., allergies reported in VAERS data).

S2.2.1. Association Rule Mining (ARM) and Self-Organizing Maps (SOMs): ARM has been applied in various disciplines [20–22] [23,24] [25] [26–28]. Irrespective of the domain of interest, triggering of one or more AEs can imply triggering of other AEs, consistent with the crosstalk between various physical AEs and perceptional indicators. ARM of AEs after each vaccine dose can be used to identify many-to-many relationships and propose a data-driven hypotheses generation technique. ARM aims at discovering common patterns that frequently occur in a large dataset of high dimensionality. ARM applies iterative algorithms to identify the occurrences of certain (related) events given that a base event (observation) has occurred. Association rules are the implications of the form $X \rightarrow Y$, where X and Y are disjoint item-sets (i.e., $X \cap Y = \phi$) denoted as antecedent and consequent of a rule, respectively. An association rule can be interpreted as "if X (e.g., a set of AEs) is categorized as most likely reported AEs after vaccination, then Y (another set of AEs) is also likely to be categorized as commonly reported AEs within the prescribed confidence (i.e., prescribed reliability of the inference of the rule). The strength of an association rule is measured by its *Support* and *Confidence* [29]. *Support* indicates the generality of the rule where higher *Support* indicates that the rule is more general and supported by large proportion of

data. *Confidence*, denoted as $Support(X \cup Y)/Support(X)$, measures the reliability of the inference made by a rule which can be interpreted as the probability of Y conditioned on $(P(Y|X))$. For a given rule $X \rightarrow Y$, higher *Confidence* indicates higher likelihood for Y to be present in the samples that contain X.

ARs in the present study were also validated with the help of SOM analysis demonstrating VAERS data distribution on 2D maps. Cluster analysis via SOMs has been demonstrated to be useful for discovering relationships in complex multidimensional datasets in cross-disciplinary areas of research and development [30–32] [33]. SOM clustering applies competitive learning, preserves topological structure of the input space, and transforms the output to a lower dimension (i.e., 2-D map of cells within SOM clusters). Through the discretized 2-D representation of multidimensional data via SOMs, preliminary feature selection can also be explored to identify AEs of significance for a predictive model development and enhancing data quality. Proximities of cells in SOMs indicate similarities among the attributes (AEs in the present study) in terms of the Euclidean or Hamming distance among data samples. The utility of SOMs for data visualization, as well as feature selection, has also been demonstrated for exploratory data analyses [25,30,31,33–38].

S2.2.2. Bipartite graphs

Interrelationships between AEs and available variables (age, vaccine manufacturer, gender, allergies) were illustrated by bipartite graphs, allowing fast identification of important subsets of factors that may show potential associations among commonly reported AEs. Interrelationships between 20 allergies, gender and 7 age groups were illustrated as bipartite graphs [39–41]. In bipartite graphs, pre-existing conditions, age groups and gender are denoted by bars (nodes) on the

right side, with the bar height proportional to the percentage of samples reporting their categories for each of the 20 most commonly reported AEs.

S3. Results based on VAERS reports without duplicate record removal

Figs. S1-2 show relative frequencies of the 20 highest reported AEs (based on uncleaned data from 905,976 VAERS reports) for all age groups per the three vaccine types (Pfizer-BioNTech, Moderna, and Janssen) and children of age up to (and inclusive of) 15 years old, respectively. Most commonly reported AEs for each manufacturer were mostly consistent as shown in **Fig. S1**. There were 13 effects {*arthralgia, asthenia, chills, dizziness, dyspnoea, fatigue, headache, injection site pain, myalgia, nausea, pain, pain in extremity, pyrexia*} that were common among the 20 highest reported AEs for all three vaccine manufacturers with {*pruritus, rash*} and {*paraesthesia, vomiting*} as additional pairs common in {Pfizer-BioNTech, Moderna} and {Pfizer-BioNTech, Janssen}, respectively. Survey data also reported {*headache, aches, chills, pain in muscle, dizziness, nausea, vomiting*, and *rash*} to be the most commonly reported AEs (**Table S2**). It is interesting to note that VAERS dataset (both with and without duplicate removal) highlighted four *injection site* related effects {*injection site (erythema, pruritus, swelling, warmth)*} to be among the top 20 AEs for Moderna. This is not surprising as also noticed in the AEs reported in survey data where 51% of the samples for Moderna reported *pain in muscle* as opposed to the same AE reported by only 39% samples for Pfizer-BioNTech (**Table S2**).

Vaccine reports for children (age groups 5 – 11 and 12 – 15) have also been reported in VAERS to assess vaccine safety for children. At the time the analysis conducted in this study (11/16/2021), a total of 15,987 reported VAERS samples (before the merger of duplicate VAERS rows) were of the age under 16 years old which were collected and analyzed separately in order to explore the

commonality between the AEs with respect to different age groups. It was aimed to discover whether there are any meaningful patterns (i.e., the reported AEs) that appear collectively in children when compared to adults, or they change as the age group progresses to an older population. Children reports from VAERS were distributed as 14,699, 1004, 268, and 16 for Pfizer-BioNTech, Moderna, Janssen, and unknown, respectively. Data cleaning other than duplicate row removal was applied for children reports where rows that reported any attribute (column) from {*age group, gender, symptom,* and *vaccine manufacturer*} as "unknown" were removed from the dataset. Accordingly, post cleaning data comprised of 13,338 reports with distribution of 12,922, 303, and 113 for Pfizer-BioNTech, Moderna, and Janssen, respectively.

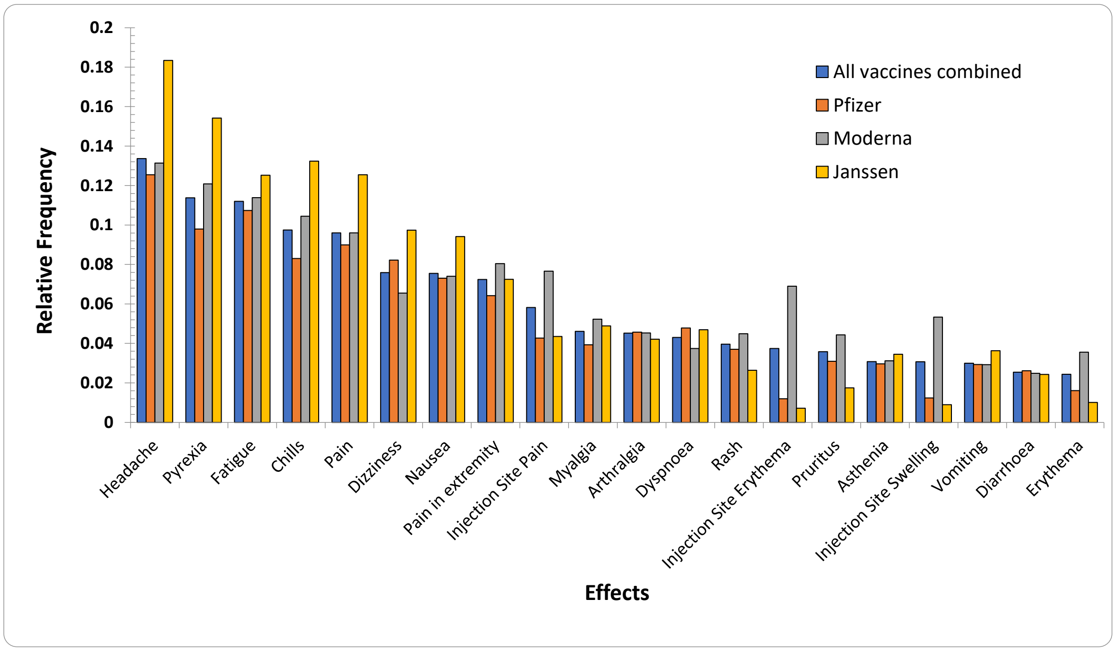

Figure S1: Relative frequencies of the top 20 AEs appeared in VAERS reports for all age groups per the three vaccine producers (Pfizer-BioNTech, Moderna, and Janssen). The subset {*chest pain, Dyspnoea, hyperhidrosis,* and *myocarditis*} was among the lowest reported effects for age group (5 – 11 years) in comparison to the effects reported for the group 12 – 15 and other 16 most commonly reported effects.

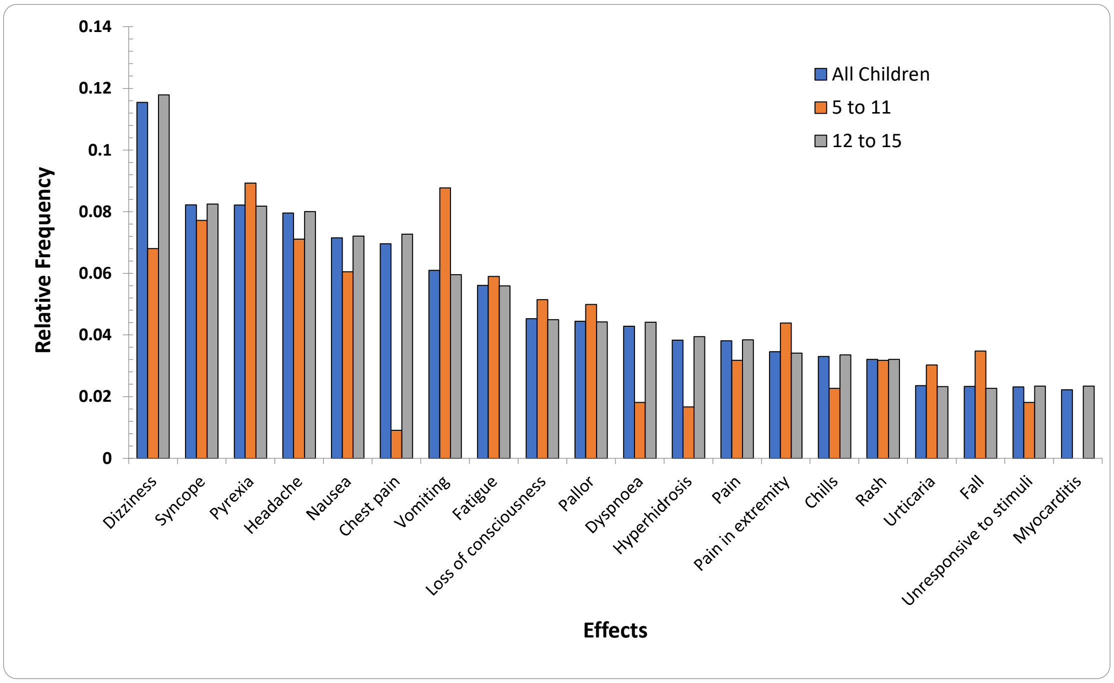

Figure S2: Relative frequencies of the top 20 AEs appeared in VAERS reports for children (discretized age groups of 5 – 11 years). The subset {*chest pain, Dyspnoea, hyperhidrosis,* and *myocarditis*} was among the lowest reported effects for age group (5 – 11 years) in comparison to the effects reported for the group 12 – 15 and other 16 most commonly reported effects.

Table S3: 20 most commonly reported AEs ranked with respect to the age groups and gender based on the percentage of VAERS samples reporting the corresponding effect (minimum 0% to maximum 15%). Heatmap cells are colored according to the percentage of reported samples and the effects are sorted according to the percentage of reported VAERS samples for age group 5 - 11

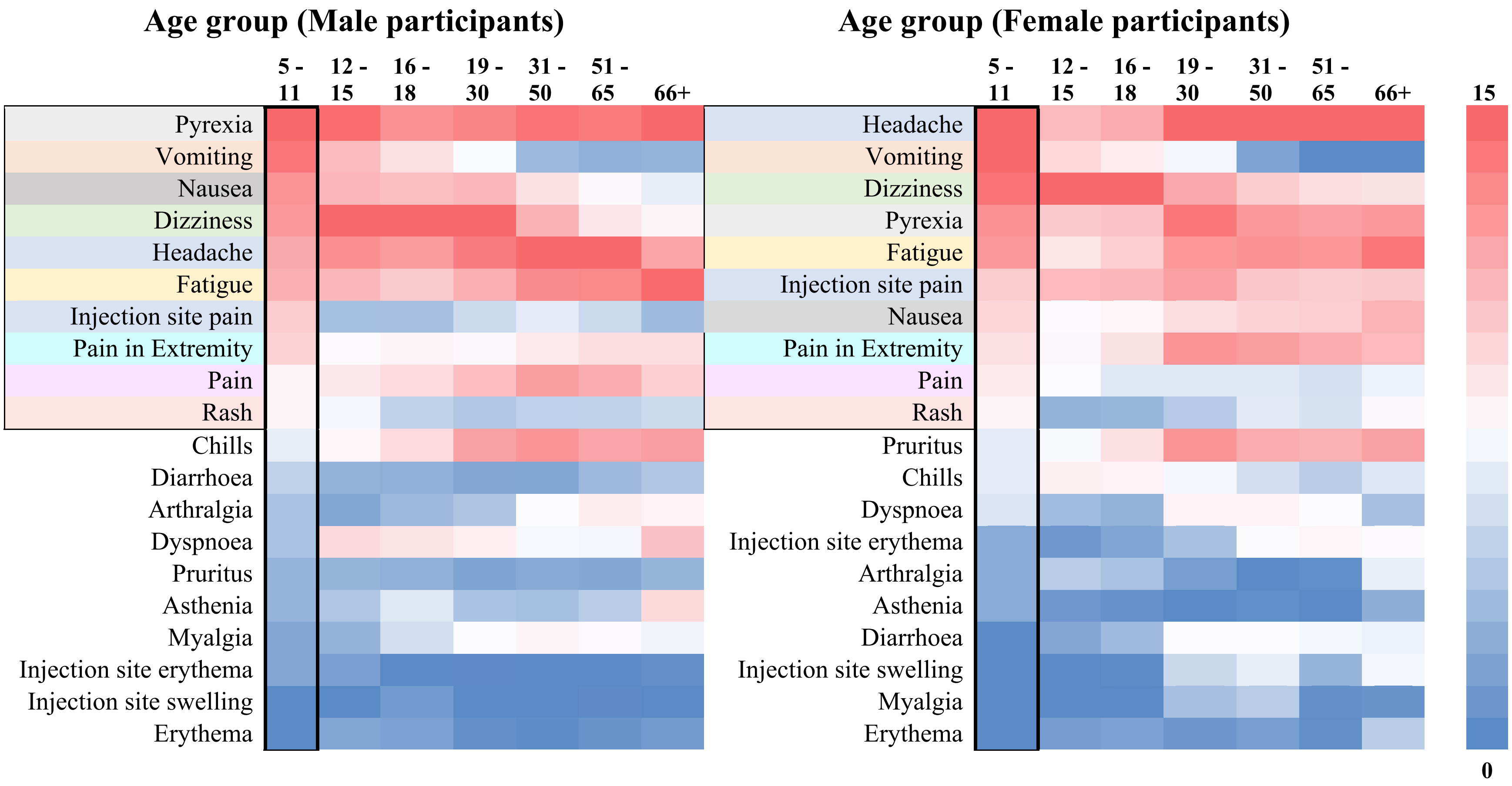

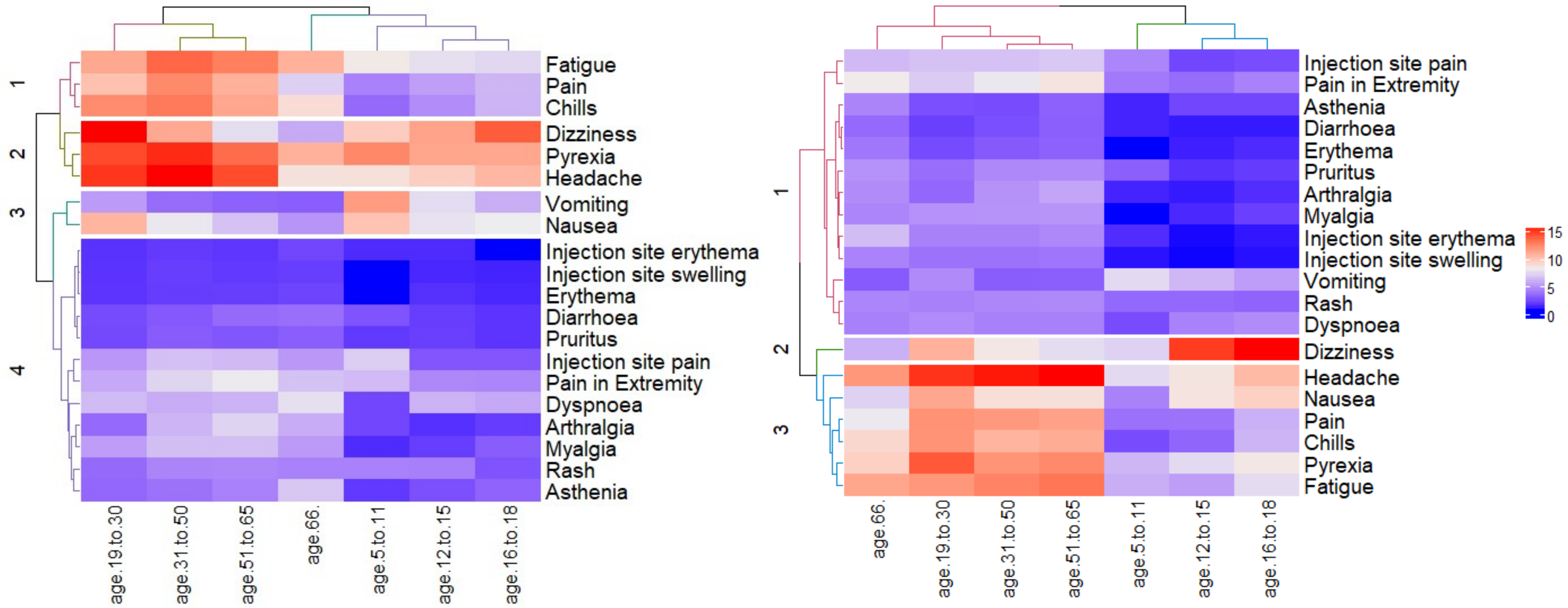

Figure S3: Hierarchical clustering of the 20 most commonly reported AEs and 7 age groups for **(a)** male and **(b)** female participants. For male participants **(a)** AEs {*pyrexia, vomiting* and *nausea*} and {*dizziness, pyrexia*} were most commonly reported for the two children age groups 5 – 11 and 12 – 15, respectively. For female participants **(b)** {*headache, pyrexia, nausea, vomiting* and *dizziness*} were the most commonly reported AEs for children age group 5 – 11 and 12 – 15, respectively with the addition of *nausea* among the 3rd most reported AE for age group 12 - 15.

S3.1. Association rules of the most commonly reported AEs via ARM and SOM

The interrelationships of post vaccine AEs from VAERS reports were analyzed via ARM for VAERS data with duplicates with respect to two major age groups (i.e., children of age up to and inclusive of 15 years old and adults aged 16 and older). For consistency and preciseness, only up to 25 top association rules with higher *lift* and *count* values were selected with diverse one-to-one and many-to-one types. Assessment of the interrelationships of AEs for children revealed 16 non-redundant rules as given in **Table S4**. From a subset of one-to-one rules, the existence of *confusional state* or *flushing* was shown to imply the existence of *dizziness* with lift over 5 (Rules R_8 and R_{10}). There was also a two-to-one association of *dizziness* with {*asthenia, hyperhidrosis*} (Rule R_{13}). Another cluster of rules indicated a many-to-one relationship of *headache* with {*chills, pain*} with the lift value of 7.1 (R_{14}). In the same cluster of rules, R_{15} and R_{16} {*chills, vomiting*} and {*pain, vomiting*} imply the existence of *pyrexia* with lift values of 9.2 and 6.8, respectively.

Interestingly, although *fatigue* appeared among the top 6 reported AEs among children when explored based on its individual frequency, its correlation with any other AE could not qualify to be among the top 16 ARs as given in **Table S4**. It is also worth noting that {*appendicectomy, appendicitis, confusional state, fall, head injury, lymph node, lymphadenopathy, troponin increased*} appeared to be strong rules although none of the above AEs were present in the 20 most commonly reported AEs in **Fig. S1**.

Table S4: Non redundant association rules for post Covid-19 vaccine AEs reported in VAERS reports for children. Rules 12 – 16 were the only non-redundant many-to-one rules identified for children. The highlighted region in gray represents a subset of rules with relatively high count in the dataset (>115) and include {*dizziness, hyperhidrosis, syncope, unresponsive to stimuli*} that were also among the 20 most commonly reported effects in children when explored based on their individual frequencies

Rule	Antecedent	Consequent	Support	Confidence	Lift	Count
R-1	Appendicectomy	Appendicitis	0.001	0.826	275.8	19
R-2	Lymph node	Lymphadenopathy	0.002	0.758	48.2	25
R-3	Rash papular	Rash	0.002	0.524	16.3	22
R-4	Rash pruritic	Rash	0.004	0.600	18.7	54
R-5	Rash erythematous	Rash	0.004	0.614	19.2	51
R-6	Body temperature	Pyrexia	0.005	0.590	7.2	62
R-7	Head injury	Fall	0.007	0.682	29.3	88
R-8	Confusional state	Dizziness	0.005	0.600	5.2	72
R-9	Flushing	Hyperhidrosis	0.010	0.720	18.8	134
R-10	Flushing	Dizziness	0.009	0.634	5.5	118
R-11	Unresponsive to stimuli	Syncope	0.014	0.602	7.3	186
R-12	Chest pain, Troponin increased	Myocarditis	0.003	0.500	22.5	39
R-13	Asthenia, Hyperhidrosis	Dizziness	0.002	0.763	6.6	29
R-14	Chills, Pain	Headache	0.002	0.564	7.1	31
R-15	Chills, Vomiting	Pyrexia	0.002	0.759	9.2	22
R-16	Pain, Vomiting	Pyrexia	0.001	0.560	6.8	14

Analysis of the ARs among post vaccine AEs was also carried out for each of the three vaccine types, as illustrated in **Tables S5-7**. For consistency and preciseness, only up to 25 top association rules with higher *lift* and *count* values were selected with diverse one-to-one and many-to-one types. Although Pfizer-BioNTech and Moderna have shown to be above 90% effective in their trial studies, it was worth noticing that 2,191 entries in the data for Pfizer-BioNTech (**Table S5**) indicated that the drug was ineffective and led to COVID-19 reported in the consequent with lift of 18.04 (R_6). It is however noted that the verification of such rules from only VAERS reports is not feasible and should only be considered for further studies with the help of quality data when made available. *Body temperature*, along with other AEs such as *headache*, *fatigue*, and *pain* implied *chills* and *pyrexia* as consequents (R_{14-17}). *Chills* in conjunction with *myalgia* and *nausea* was resulted in reporting *headache* as the consequent ($R_{19,20}$). *Injection site pruritus* and *injection site swelling* implied *injection site erythema* (R_1) with highest *lift* value of 52 and *count* value of 4,572, where *chills* → *pyrexia (R3)* had the highest *count* of 570.

Consistent with Pfizer-BioNTech, *headache* appeared in the consequent of 15 out of 25 rules for Moderna (**Table 4 –** manuscript), as opposed to only 4 rules reporting *headache* in the consequent for VAERS data with duplicates (**Table S6**). In addition to the similar distributions of the rules ($R_{5,6,8,10-16}$) showed for Pfizer-BioNTech, rules (R_{9-25}) for Moderna indicated discernible patterns in the component planes for the set of AEs {*arthralgia, chills, fatigue, headache, myalgia, nausea, pain, pyrexia*} as given in **Fig. S5**. It was interesting to note that, in **Table S6**, 8 out 17 rules for Moderna vaccine reported *injection site* related effects (e.g., injection site {*inflammation, pruritus, pain, induration, mass, warmth, swelling, erythema*}) in either the antecedent or the consequent of the rules, which is consistent with the observations from simple exploratory analysis. Reports for Moderna consisted of *injection site* related AEs with significantly larger percentage than that of

Pfizer-BioNTech. *Injection site pruritus* → *injection site erythema* (R_9) had the highest *count* of 12,717 for Moderna. **Table S7** shows ARs for Janssen, where although 12 of 20 most commonly reported AEs were present (**Fig. S1**), 13 out 25 ARs for Janssen reported *headache* in the consequent with {*fatigue, chills, myalgia, nausea*} appearing most commonly in the antecedent. The rule {*chills, fatigue*} → *headache* had the highest count of 1,815 (R_{25}), where Rule R_1 indicated a noteworthy association of {*vaccination breakthrough infection* → *COVID-19*} with 103 *count* value.

Table S5: Non redundant association rules for post Covid-19 vaccine effects reported in VAERS reports for Pfizer-BioNTech vaccine. Rules 10 - 20 were the non-redundant many-to-one rules identified for Pfizer-BioNTech. The highlighted regions in gray represent the subset of rules with relatively high count in the dataset (>1000). The rules below include {*dizziness, pyrexia, headache, nausea, vomiting, chills, rash, injection site pain, myalgia*} that were also among the 20 most commonly reported effects for VAERS reports for Pfizer-BioNTech when explored based on their individual frequencies.

Rule	Antecedent	Consequent	Support	Confidence	Lift	Count
R-1	Vaccination failure	COVID-19	0.0013	0.77	17.00	528
R-2	Vaccine breakthrough infection	COVID-19	0.0014	0.52	11.53	548
R-3	Acute respiratory failure	COVID-19	0.0011	0.52	11.53	435
R-4	Head injury	Fall	0.0013	0.57	71.51	501
R-5	COVID-19 pneumonia	COVID-19	0.0022	0.58	12.88	861
R-6	Drug ineffective	COVID-19	0.0055	0.82	18.04	2191
R-7	Heart rate	Heart rate increased	0.0026	0.53	33.17	1033
R-8	Lymph node pain	Lymphadenopathy	0.0041	0.68	25.09	1629
R-9	Rash pruritic	Rash	0.0038	0.52	13.96	1527
R-10	Injection site swelling, Injection site warmth	Injection site erythema	0.0011	0.55	46.01	460
R-11	Injection site pruritus, Injection site swelling	Injection site erythema	0.0014	0.63	52.78	570
R-12	Injection site pain, Injection site pruritus	Injection site erythema	0.0010	0.56	46.70	406
R-13	Flushing, Hyperhidrosis	Dizziness	0.0015	0.55	6.72	602
R-14	Body temperature, Pain	Pyrexia	0.0013	0.57	5.79	529
R-15	Body temperature, Pain	Chills	0.0012	0.50	6.06	469
R-16	Body temperature, Fatigue	Chills	0.0018	0.50	6.08	736
R-17	Body temperature, Headache	Chills	0.0021	0.51	6.16	857
R-18	Headache, Vomiting	Nausea	0.0013	0.51	6.92	533
R-19	Chills, Myalgia	Headache	0.0041	0.52	4.12	1644
R-20	Chills, Nausea	Headache	0.0054	0.51	4.06	2155

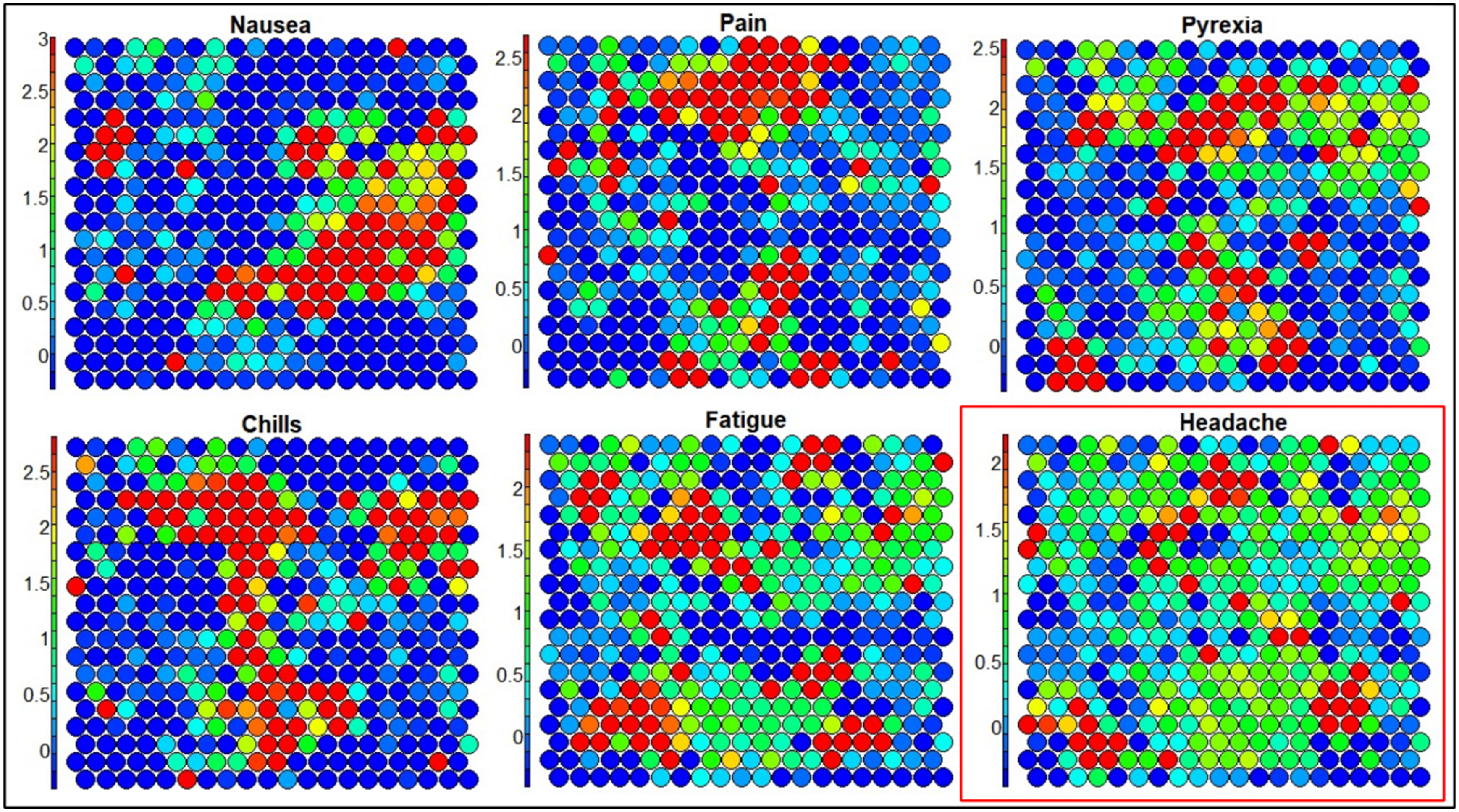

Figure S4: SOM analysis of the top 20 most reported effects from the reports filtered for Pfizer-BioNTech vaccine. Association of the effect *Headache* is shown with {*chills, fatigue, nausea, pain, pyrexia*} in the form of 2D cluster similarities is demonstrated as also shown in the rules *R9-16,19-25* in **Table S5**.

Table S6: Non redundant association rules for post Covid-19 vaccine effects reported in VAERS reports for Moderna vaccine. Rules 11 - 17 were the non-redundant many-to-one rules identified for Moderna. The highlighted regions in gray represent the subset of rules with relatively high count in the dataset (>1000). The rules below include {*injection site erythema, pyrexia, headache, nausea, vomiting, chills, rash, injection site pain, myalgia*} that were also among the 20 most commonly reported effects for VAERS reports for Pfizer-BioNTech when explored based on their individual frequencies.

Rule	Antecedent	Consequent	Support	Confidence	Lift	Count
R-1	Product temperature excursion issue	Poor quality product administered	0.001	0.82	213.221704	609
R-2	Product temperature excursion issue	Product storage error	0.001	0.81	84.070279	607
R-3	COVID-19 pneumonia	COVID-19	0.001	0.55	22.442675	463
R-4	Injection site inflammation	Injection site erythema	0.001	0.57	8.300623	460
R-5	Lymph node pain	Lymphadenopathy	0.003	0.57	28.288911	1065
R-6	Injection site mass	Injection site erythema	0.003	0.53	7.699477	1063
R-7	Injection site induration	Injection site erythema	0.009	0.64	9.278879	3611
R-8	Injection site warmth	Injection site erythema	0.019	0.53	7.737559	7897
R-9	Injection site pruritus	Injection site erythema	0.031	0.63	9.130441	12717
R-10	Injection site swelling	Injection site erythema	0.029	0.54	7.840362	11727
R-11	Body temperature increased, Fatigue	Chills	0.001	0.51	4.843717	538
R-12	Pruritus, Skin warm	Erythema	0.002	0.51	14.399942	831
R-13	Fatigue, Hyperhidrosis	Chills	0.002	0.50	4.806119	804
R-14	Arthralgia, Nausea	Headache	0.001	0.50	3.804697	551
R-15	Chills, Nausea	Headache	0.007	0.51	3.884926	2978
R-16	Arthralgia, Chills, Myalgia	Headache	0.001	0.51	3.902462	479
R-17	Chills, Fatigue, Injection site pain	Headache	0.002	0.51	3.854109	741

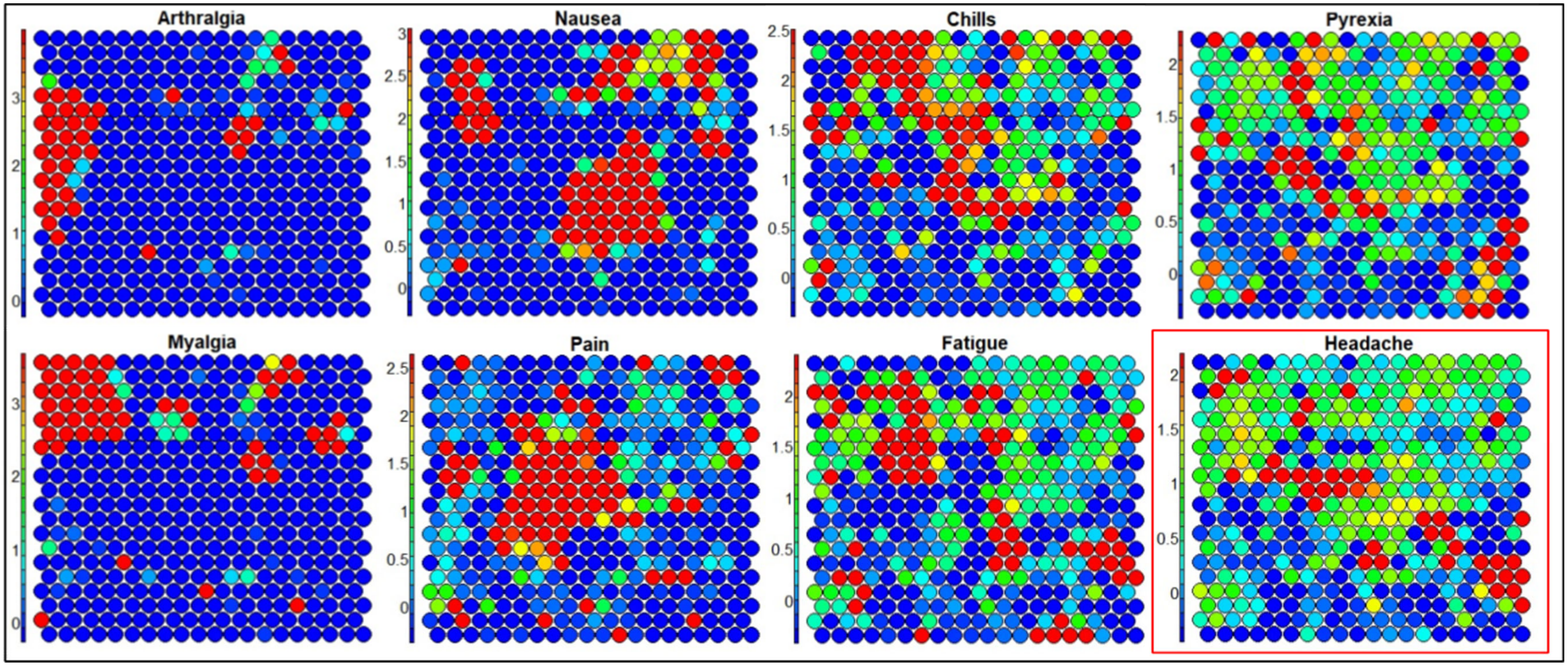

Figure S5: SOM analysis of the top 20 most reported effects from the reports filtered for Moderna vaccine. Association of the effect *Headache* is shown with {*arthralgia, chills, fatigue, myalgia, nausea, pain, pyrexia*} in the form of 2D cluster similarities is demonstrated as also shown in the rules $R_{9\text{-}16,19\text{-}25}$ in **Table S6**.

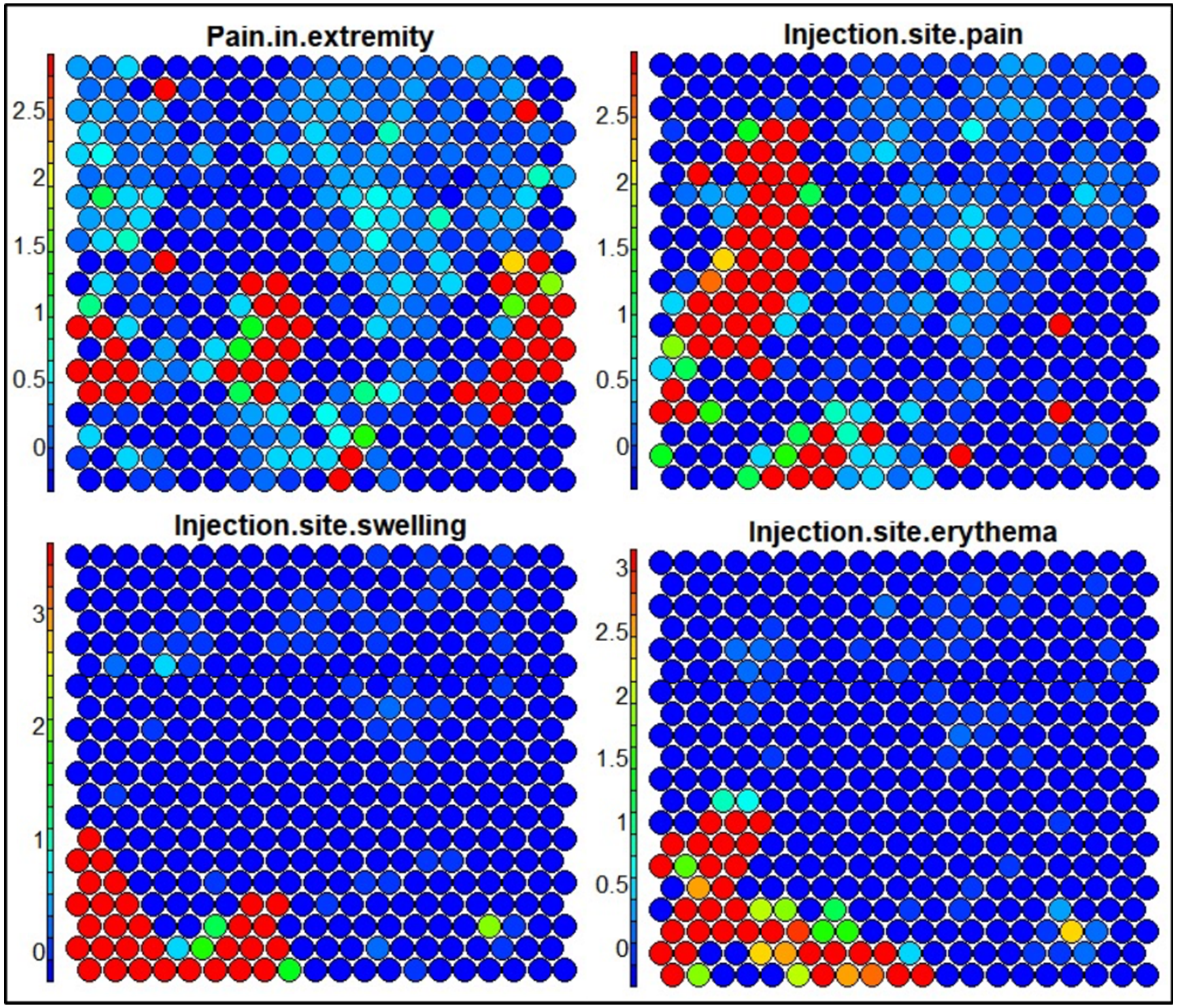

Figure S6: SOM analysis of the top 20 most reported effects from the reports filtered for Moderna vaccine. Separate class of AEs related to *injection site* identified by ARM and SOM for Moderna. Rule *R*5 indicated the correlation of *injection site swelling* and *erythema* as also shown in the bottom row in the above Figure.

Table S7: Non redundant association rules for post Covid-19 vaccine effects reported in VAERS reports for Janssen vaccine. Rules 9 - 25 were the non-redundant many-to-one rules identified for Janssen. The highlighted regions in gray represent the subset of rules with relatively high count in the dataset (>500). The rules below include {*injection site erythema, injection site swelling, pyrexia, headache, nausea, chills, rash, injection site pain, pain, fatigue, myalgia*} that were also among the 20 most commonly reported effects for VAERS reports for Janssen when explored based on their individual frequencies.

Rule	Antecedent	Consequent	Support	Confidence	Lift	Count
R-1	Vaccine breakthrough infection	COVID-19	0.0012	0.52	19.24	103
R-2	Antibody test	Therapy non-responder	0.0012	0.58	172.14	98
R-3	Poor quality product administered	Product storage error	0.0031	0.62	85.99	254
R-4	COVID-19 pneumonia	COVID-19	0.0019	0.57	21.46	154
R-5	Head injury	Fall	0.0020	0.57	54.04	168
R-6	Rash erythematous	Rash	0.0021	0.50	19.10	177
R-7	Rash pruritic	Rash	0.0028	0.54	20.36	229
R-8	Body temperature increased	Chills	0.0046	0.51	3.84	380
R-9	Injection site erythema, Injection site swelling	Injection site pain	0.0015	0.51	11.73	122
R-10	Abdominal pain upper, Fatigue	Headache	0.0011	0.52	2.82	90
R-11	Fatigue, Insomnia	Headache	0.0011	0.53	2.87	89
R-12	Chills, Influenza like illness	Headache	0.0018	0.54	2.93	149
R-13	Decreased appetite, Headache	Fatigue	0.0023	0.54	4.30	190
R-14	Chills, Heart rate increased	Headache	0.0014	0.50	2.74	120
R-15	Pain, Tremor	Pyrexia	0.0016	0.54	3.53	134
R-16	Body temperature, Pain	Pyrexia	0.0018	0.60	3.91	146
R-17	Chills, Injection site pain	Headache	0.0044	0.52	2.85	368
R-18	Fatigue, Hyperhidrosis	Headache	0.0028	0.50	2.75	237
R-19	Chills, Hyperhidrosis	Headache	0.0048	0.53	2.88	398
R-20	Dizziness, Myalgia	Headache	0.0012	0.51	2.80	98
R-21	Fatigue, Myalgia	Headache	0.0065	0.53	2.89	542
R-22	Chills, Myalgia	Headache	0.0069	0.57	3.12	577
R-23	Fatigue, Nausea	Headache	0.0081	0.54	2.94	672
R-24	Chills, Nausea	Headache	0.0104	0.56	3.06	869
R-25	Chills, Fatigue	Headache	0.0218	0.50	2.74	1815

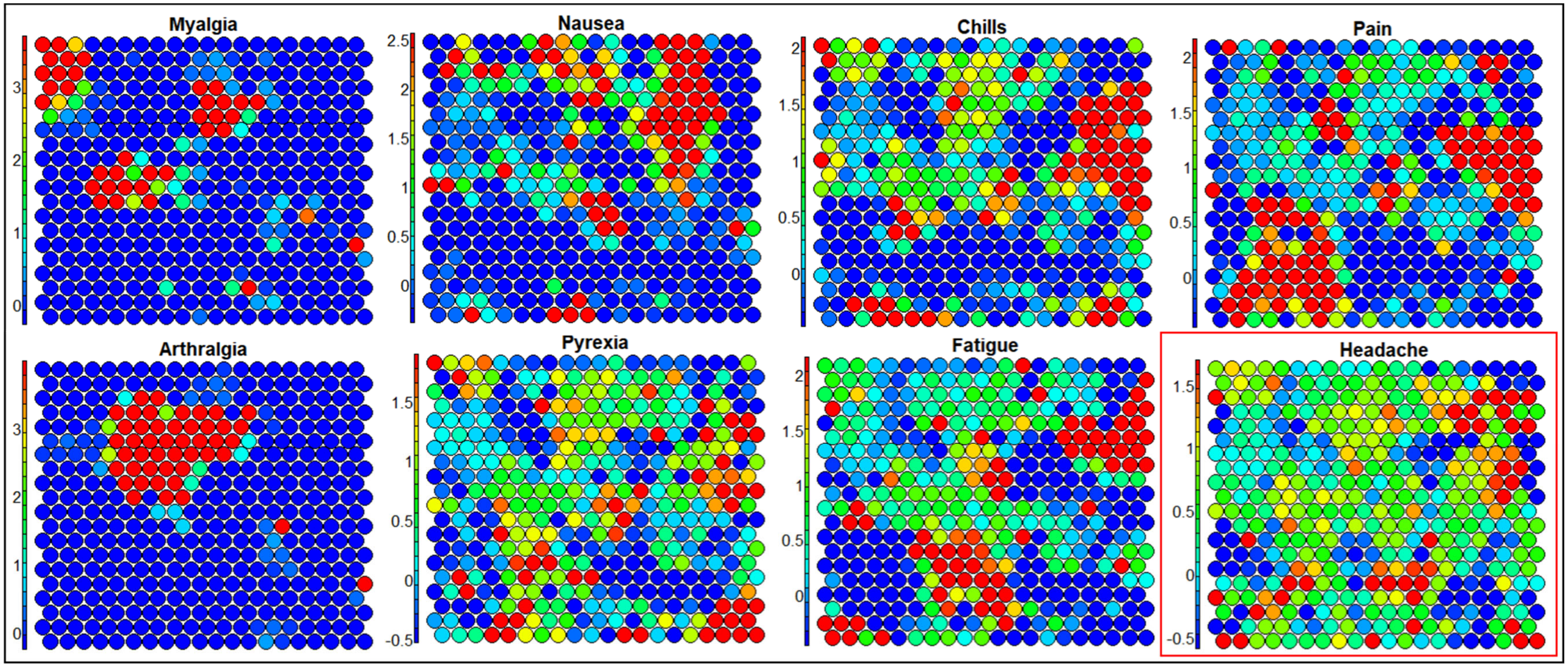

Figure S7: SOM analysis of the top 20 most reported effects from the reports filtered for Janssen vaccine. Association of the effect *Headache* is shown with {*arthralgia, chills, fatigue, myalgia, nausea, pain, pyrexia*} in the form of 2D cluster similarities is demonstrated as also shown in the rules *R4,5,7-10* in **Table 4 (manuscript)**

S3.2 Interrelations of side effects via bipartite graphs

The established bipartite graphs in the present study can be useful for inspecting the existence of the side effects relative to age groups and gender as illustrated in **Fig. S8**. In the bipartite graphs (**Fig. 5** - manuscript), the distribution of AEs (right side) was identified as percentage appearance of each AE per vaccine in VAERS data along with the prior distributions of three vaccines (left side). In the bipartite graph (**Fig. 5**), AEs are denoted by the bars (nodes) on the right side, with the bar height proportional to their reported frequency in VAERS data over the 3 vaccines. Each bar for AE is further split into sub-bars representing its distribution (in terms of the percent appearance in VAERS reports) across 3 vaccines. The left side bars of the bipartite graphs (**Fig. S8**) identify the 3 vaccines with the bar height indicating the percentage of total VAERS data reporting each vaccine.

Bipartite graph for the existence of AEs for the age groups (**Fig. S9a**) showed highest percentage of reports (36%) associated with the age group of 31 – 50 years old followed by the age group of 51 – 65 years old to be of 28% of the VAERS reports. Bipartite graphs (**Figs. 5** (manuscript) and **S8-9**) allow bidirectional exploration of the VAERS data for detailed information about a specific AE (i.e., vaccine → side effect) or an AE for different age groups or genders (i.e., age group → side effect, or gender → side effect). For example, in the direction of gender → side effect, focusing the bipartite graph of *gender* (**Fig. S9b**) on *headache* and *fatigue* revealed that, 77% of the VAERS reports were for *female* with AEs *headache, fatigue* and *pyrexia* to be reported with highest percentages of 11%, 9%, and 9%, respectively. In addition to the above bipartite graphs, one can also explore the reported AEs and their interrelationships with pre-existing conditions (i.e., allergies column in VAERS data) (allergy → side effect) as illustrated in **Fig. 5e** (manuscript). This bidirectional exploration using bipartite graphs can be conducted along the hierarchy of these variables to identify informative interrelationships of AEs with different allergies for both genders

and at different levels of age. The above bidirectional explorations can be helpful to interrogate causal relationships based on quality vaccine data, in particular when the data from Covid-19 vaccine producers becomes available.

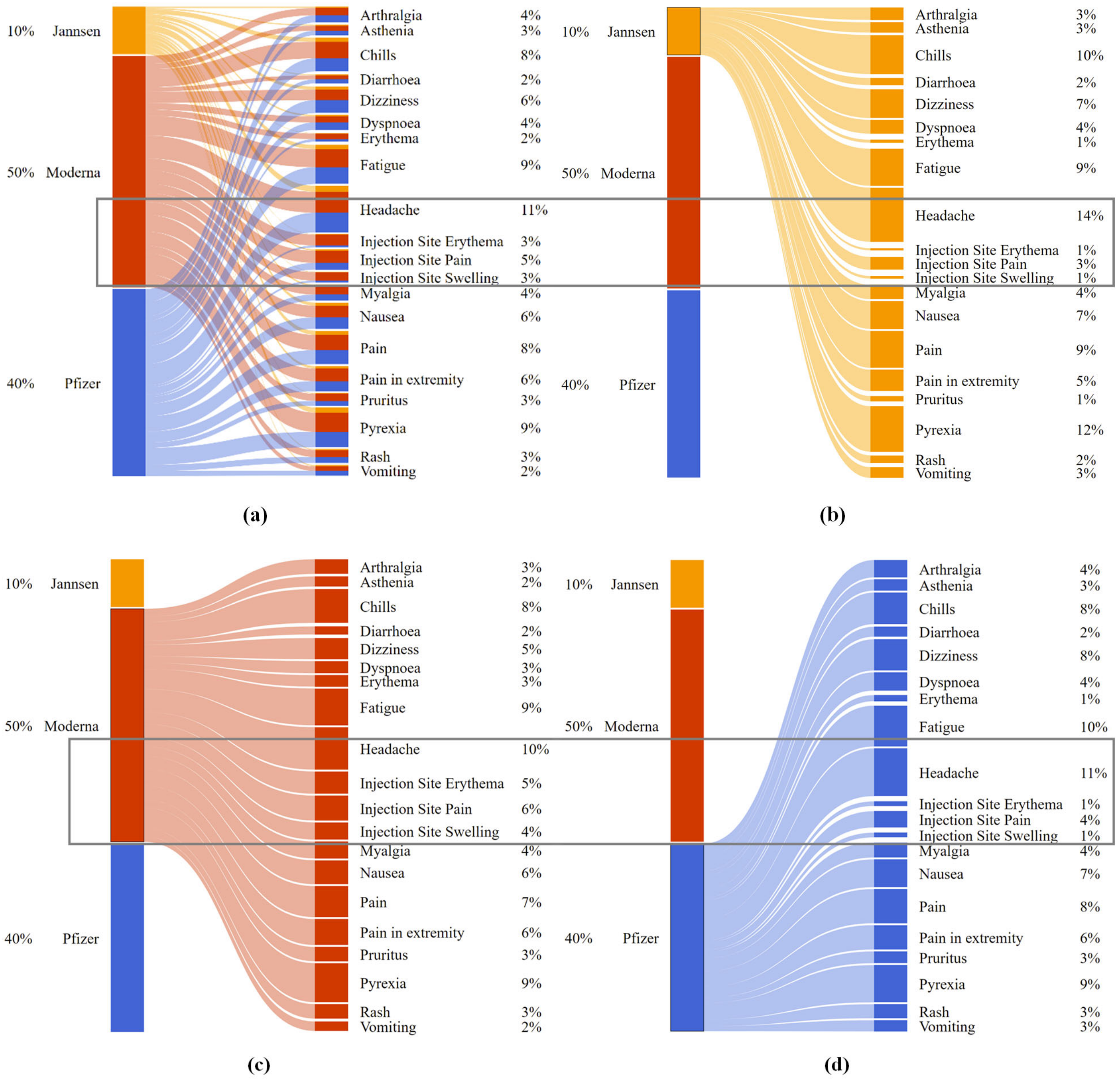

Figure S8: Bipartite graphs for the correlations of 20 most commonly reported AEs with the 3 vaccine producers (Pfizer-BioNTech, Moderna, and Janssen). **Figure (a)** shows the distribution of the entire VAERS dataset with respect to the vaccine producers, and **b – d** show the distributions of AEs with respect to each vaccine.

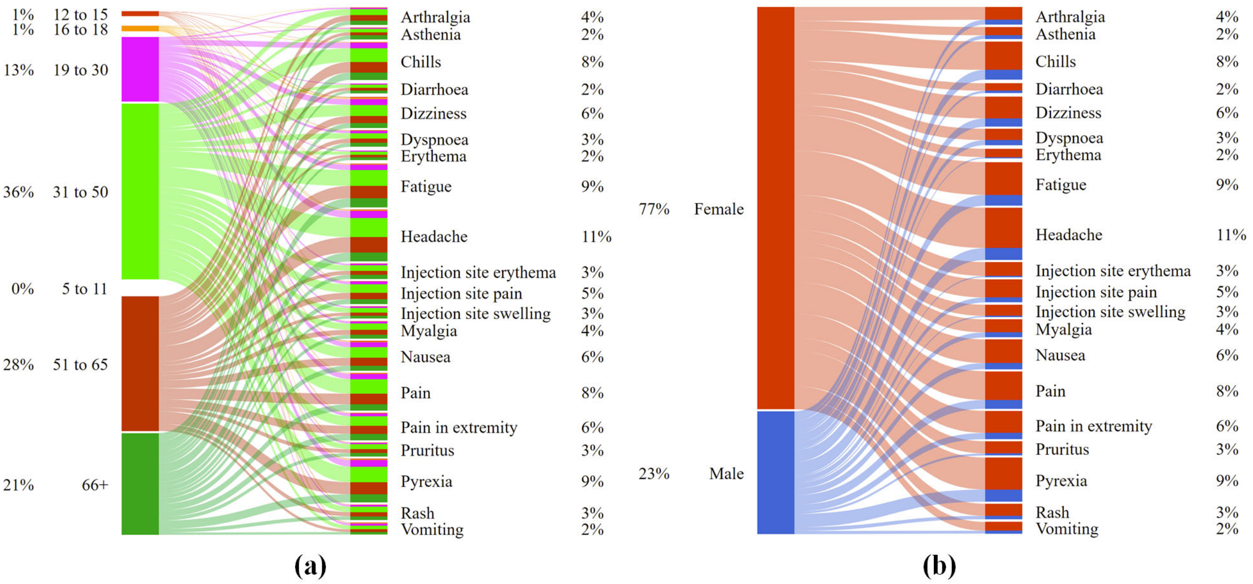

Figure S9: Bipartite graphs for the correlations of 20 most commonly reported effects with different age groups (**a**) and genders (**b**). From the graph of age group, the groups 31 – 50 and 51 – 65 appear to be the highest reported groups for which the VAERS reports were submitted, whereas among all VAERS reports, 77% of them indicated gender female as shown in **(b)**

References

1. Su JR. Myopericarditis following COVID-19 vaccination : updates from the Vaccine Adverse Event Reporting System (VAERS) [Internet]. Team. CDCC-19 VTFVS, editor. Atanta, GA; (ACIP meeting COVID-19 Vaccines; vol. 202113). Available from: https://stacks.cdc.gov/view/cdc/110920
2. Myers TR, McNeil MM, Ng CS, Li R, Marquez PL, Moro PL, et al. Adverse events following quadrivalent meningococcal diphtheria toxoid conjugate vaccine (Menactra®) reported to the Vaccine Adverse Event Reporting System (VAERS), 2005–2016. Vaccine. 2020;
3. Luo C, Jiang Y, Du J, Tong J, Huang J, Lo Re V, et al. Prediction of post-vaccination Guillain-Barré syndrome using data from a passive surveillance system. Pharmacoepidemiol Drug Saf. 2021;
4. Miller NZ. Vaccines and sudden infant death: An analysis of the VAERS database 1990–2019 and review of the medical literature. Toxicol Reports. 2021;
5. Baker MA, Kaelber DC, Bar-Shain DS, Moro PL, Zambarano B, Mazza M, et al. Advanced Clinical Decision Support for Vaccine Adverse Event Detection and Reporting. Clin Infect Dis. 2015;
6. Sukumaran L, McNeil MM, Moro PL, Lewis PW, Winiecki SK, Shimabukuro TT. Adverse events following measles, mumps, and rubella vaccine in adults reported to the

vaccine adverse event reporting system (VAERS), 2003-2013. Clin Infect Dis. 2015;
7. Moro PL, Woo EJ, Paul W, Lewis P, Petersen BW, Cano M. Post-Marketing Surveillance of Human Rabies Diploid Cell Vaccine (Imovax) in the Vaccine Adverse Event Reporting System (VAERS) in the United States, 1990–2015. PLoS Negl Trop Dis. 2016;
8. Loughlin AM, Marchant CD, Adams W, Barnett E, Baxter R, Black S, et al. Causality assessment of adverse events reported to the Vaccine Adverse Event Reporting System (VAERS). Vaccine. 2012;
9. Myers TR, McNeil MM, Ng CS, Li R, Lewis PW, Cano M V. Adverse events following quadrivalent meningococcal CRM-conjugate vaccine (Menveo®) reported to the Vaccine Adverse Event Reporting system (VAERS), 2010–2015. Vaccine. 2017;
10. Gatti M, Raschi E, Moretti U, Ardizzoni A, Poluzzi E, Diemberger I. Influenza vaccination and myo-pericarditis in patients receiving immune checkpoint inhibitors: Investigating the likelihood of interaction through the vaccine adverse event reporting system and vigibase. Vaccines. 2021;
11. VAERS. VAERS Data. https://vaers.hhs.gov/data/datasets.html? 2021.
12. Miller ER, McNeil MM, Moro PL, Duffy J, Su JR. The reporting sensitivity of the Vaccine Adverse Event Reporting System (VAERS) for anaphylaxis and for Guillain-Barré syndrome. Vaccine. 2020;
13. Botsis T, Nguyen MD, Woo EJ, Markatou M, Ball R. Text mining for the Vaccine Adverse Event Reporting System: medical text classification using informative feature selection. J Am Med Informatics Assoc [Internet]. 2011 Sep 1;18(5):631–8. Available from: https://doi.org/10.1136/amiajnl-2010-000022
14. Du J, Xiang Y, Sankaranarayanapillai M, Zhang M, Wang J, Si Y, et al. Extracting postmarketing adverse events from safety reports in the vaccine adverse event reporting system (VAERS) using deep learning. J Am Med Informatics Assoc [Internet]. 2021 Jul 1;28(7):1393–400. Available from: https://doi.org/10.1093/jamia/ocab014
15. Lian AT, Du J, Tang L. Using a Machine Learning Approach to Monitor COVID-19 Vaccine Adverse Events (VAE) from Twitter Data. Vaccines. 2022 Jan;10(1).
16. Sujatha R, Krishna BVS, Chatterjee JM, Naidu PR, Jhanjhi NZ, Charita C, et al. Prediction of Suitable Candidates for COVID-19 Vaccination. Vol. 32, Intelligent Automation \& Soft Computing . 2022.
17. Xie J, Zhao L, Zhou S, He Y. Statistical and Ontological Analysis of Adverse Events Associated with Monovalent and Combination Vaccines against Hepatitis A and B Diseases. Sci Rep [Internet]. 2016;6(1):34318. Available from: https://doi.org/10.1038/srep34318
18. Miller ER, Lewis P, Shimabukuro TT, Su J, Moro P, Woo EJ, et al. Post-licensure safety surveillance of zoster vaccine live (Zostavax®) in the United States, Vaccine Adverse Event Reporting System (VAERS), 2006–2015. Hum Vaccin Immunother [Internet]. 2018 Aug 3;14(8):1963–9. Available from: https://doi.org/10.1080/21645515.2018.1456598
19. Du J, Xiang Y, Sankaranarayanapillai M, Zhang M, Wang J, Si Y, et al. Extracting postmarketing adverse events from safety reports in the vaccine adverse event reporting system (VAERS) using deep learning. J Am Med Informatics Assoc. 2021 Jul;28(7):1393–400.
20. Oellrich A, Jacobsen J, Papatheodorou I, Smedley D. Using association rule mining to determine promising secondary phenotyping hypotheses. Bioinformatics [Internet]. 2014 Jun 15;30(12):i52–9. Available from: http://dx.doi.org/10.1093/bioinformatics/btu260

21. Naulaerts S, Meysman P, Bittremieux W, Vu TN, Vanden Berghe W, Goethals B, et al. A primer to frequent itemset mining for bioinformatics. Brief Bioinform [Internet]. 2015 Mar 26;16(2):216–31. Available from: http://www.ncbi.nlm.nih.gov/pmc/articles/PMC4364064/
22. Guzzi PH, Milano M, Cannataro M. Mining Association Rules from Gene Ontology and Protein Networks: Promises and Challenges. Procedia Comput Sci [Internet]. 2014;29:1970–80. Available from: http://www.sciencedirect.com/science/article/pii/S1877050914003585
23. Park SH, Reyes JA, Gilbert DR, Kim JW, Kim S. Prediction of protein-protein interaction types using association rule based classification. BMC Bioinformatics [Internet]. 2009;10(1):36. Available from: http://dx.doi.org/10.1186/1471-2105-10-36
24. Nafar Z, Golshani A. Data Mining Methods for Protein-Protein Interactions. 2006 Canadian Conference on Electrical and Computer Engineering. Ottawa, ON, Canada. 07-10 May 2006: IEEE; 2006. p. 991–4.
25. Liu R, France B, George S, Rallo R, Zhang H, Xia T, et al. Association rule mining of cellular responses induced by metal and metal oxide nanoparticles. Analyst [Internet]. 2014;139(5):943–53. Available from: http://dx.doi.org/10.1039/C3AN01409F
26. Mallik S, Mukhopadhyay A, Maulik U, Bandyopadhyay S. Integrated analysis of gene expression and genome-wide DNA methylation for tumor prediction: An association rule mining-based approach. 2013 IEEE Symposium on Computational Intelligence in Bioinformatics and Computational Biology (CIBCB). Singapore. 16-19 April 2013: IEEE; 2013. p. 120–7.
27. Martinez R, Pasquier N, Pasquier C. GenMiner: mining non-redundant association rules from integrated gene expression data and annotations. Bioinformatics [Internet]. 2008 Nov 15;24(22):2643–4. Available from: http://dx.doi.org/10.1093/bioinformatics/btn490
28. Alves R, Rodriguez-Baena DS, Aguilar-Ruiz JS. Gene association analysis: a survey of frequent pattern mining from gene expression data. Brief Bioinform [Internet]. 2010 Mar 1;11(2):210–24. Available from: http://dx.doi.org/10.1093/bib/bbp042
29. Zhang C, Zhang S. Association Rule Mining: Models and Algorithms. Berlin, Heidelberg: Springer-Verlag; 2002.
30. Chon TS. Self-Organizing Maps applied to ecological sciences. Vol. 6, Ecological Informatics. 2011. p. 50–61.
31. Tamayo P, Slonim D, Mesirov J, Zhu Q, Kitareewan S, Dmitrovsky E, et al. Interpreting patterns of gene expression with self-organizing maps: methods and application to hematopoietic differentiation. Proc Natl Acad Sci U S A. 1999;96(6):2907–12.
32. Törönen P, Kolehmainen M, Wong G, Castrén E. Analysis of gene expression data using self-organizing maps. FEBS Lett [Internet]. 1999;451(2):142–6. Available from: http://www.ncbi.nlm.nih.gov/pubmed/18003033
33. Bullinaria JA. Self Organizing Maps: Fundamentals [Internet]. 2004 [cited 2017 Jun 3]. Available from: http://www.cs.bham.ac.uk/~jxb/NN/l16.pdf
34. Dettmer J, Benavente R, Cummins PR, Sambridge M. Trans-dimensional finite-fault inversion. Geophys J Int [Internet]. 2014 Nov 1;199(2):735–51. Available from: http://dx.doi.org/10.1093/gji/ggu280
35. Giralt F, Espinosa G, Arenas A, Ferre-Gine J, Amat L, Gironés X, et al. Estimation of infinite dilution activity coefficients of organic compounds in water with neural classifiers. AIChE J. 2004 May;50(6):1315–43.

36. Liu R, Lin S, Rallo R, Zhao Y, Damoiseaux R, Xia T, et al. Automated Phenotype Recognition for Zebrafish Embryo Based In Vivo High Throughput Toxicity Screening of Engineered Nano-Materials. PLoS One [Internet]. 2012 Apr 10;7(4):e35014. Available from: http://dx.doi.org/10.1371%2Fjournal.pone.0035014
37. Rallo R, France B, Liu R, Nair S, George S, Damoiseaux R, et al. Self-Organizing Map Analysis of Toxicity-Related Cell Signaling Pathways for Metal and Metal Oxide Nanoparticles. Environ Sci Technol [Internet]. 2011 Feb 15;45(4):1695–702. Available from: http://www.ncbi.nlm.nih.gov/pmc/articles/PMC4418424/
38. Rhodes BC, Mahaffey JA, Cannady JD. Multiple self-organizing maps for intrusion detection. Proc 23rd …. 2000;16–9.
39. Dormann CF, Strauss R. A method for detecting modules in quantitative bipartite networks. Methods Ecol Evol. 2014;5:90–8.
40. Dormann CF, Fründ J, Blüthgen N, Gruber B. Indices, Graphs and Null Models: Analyzing Bipartite Ecological Networks. Open Ecol J. 2009;2:7–24.
41. Dormann CF, Gruber B, Fründ J. Introducing the bipartite package: analysing ecological networks. Interaction. 2008;1:0–2413793.
